# Quantifying Genomic Imprinting at Tissue and Cell Resolution in the Brain

**DOI:** 10.3390/epigenomes4030021

**Published:** 2020-09-04

**Authors:** Annie Varrault, Emeric Dubois, Anne Le Digarcher, Tristan Bouschet

**Affiliations:** 1 Institut de Génomique Fonctionnelle (IGF), Univ. Montpellier, CNRS, INSERM, 34094 Montpellier, France; annie.varrault@igf.cnrs.fr (A.V.); anne.le-digarcher@igf.cnrs.fr (A.L.D.); 2 Montpellier GenomiX (MGX), Univ. Montpellier, CNRS, INSERM, 34094 Montpellier, France; emeric.dubois@mgx.cnrs.fr

**Keywords:** genomic imprinting, allele-specific RNA-seq, scRNA-seq, brain

## Abstract

Imprinted genes are a group of ~150 genes that are preferentially expressed from one parental allele owing to epigenetic marks asymmetrically distributed on inherited maternal and paternal chromosomes. Altered imprinted gene expression causes human brain disorders such as Prader-Willi and Angelman syndromes and additional rare brain diseases. Research data principally obtained from the mouse model revealed how imprinted genes act in the normal and pathological brain. However, a better understanding of imprinted gene functions calls for building detailed maps of their parent-of-origin-dependent expression and of associated epigenetic signatures. Here we review current methods for quantifying genomic imprinting at tissue and cell resolutions, with a special emphasis on methods to detect parent-of-origin dependent expression and their applications to the brain. We first focus on bulk RNA-sequencing, the main method to detect parent-of-origin-dependent expression transcriptome-wide. We discuss the benefits and caveats of bulk RNA-sequencing and provide a guideline to use it on F1 hybrid mice. We then review methods for detecting parent-of-origin-dependent expression at cell resolution, including single-cell RNA-seq, genetic reporters, and molecular probes. Finally, we provide an overview of single-cell epigenomics technologies that profile additional features of genomic imprinting, including DNA methylation, histone modifications and chromatin conformation and their combination into sc-multimodal omics approaches, which are expected to yield important insights into genomic imprinting in individual brain cells.

## 1. Genomic Imprinting: A Prototype of Epigenetic Regulation

In mammals, the vast majority of genes are biallelically expressed: They are expressed at relatively similar levels from both the maternal and the paternal alleles on average. By contrast, a minority of genes are monoallelically expressed. The choice of the expressed allele can be random as observed for X-chromosome inactivation in adult females, olfactory receptors and random monoallelic expression (RMAE) [1,2,3]. By contrast, the choice of the expressed allele is deterministic for imprinted genes: some imprinted genes are systematically expressed from the maternal allele, while others are systematically expressed from the paternal allele [4,5,6]. To date, ~150 imprinted genes have been identified in human or mouse, including X-linked imprinted genes [7] and genes coding for microRNAs (miRs) [8]. Web resources that list imprinted genes can be found in [9,10,11].

The parent-of-origin-dependent expression of imprinted genes results from epigenetic marks, chiefly DNA methylation, which are differentially deposited (imprinted) on Imprinting Control Regions (ICRs) in egg and sperm during female and male gametogenesis [4,5,6]. In the zygote, this results in the asymmetrical distribution of methylation marks, some chromosomal regions being exclusively methylated on the maternally inherited allele and others on the paternally inherited allele. These Differentially Methylated Regions (DMRs) control the parent-of-origin-dependent expression of nearby imprinted genes, which most of the time are organized into clusters [4,5,6]. A recent study reports on the existence of an atypical form of germline genomic imprinting in the oocyte that does not depend on DNA methylation but on H3K27me3 histone modification [12]. The parent-of-origin-dependent expression of genes affected by this unusual form of genomic imprinting is transient, in contrast to classical genomic imprinting (dependent on DNA methylation) that is rather stable during lifetime for most imprinted genes [12].

In addition to DNA methylation, the parental expression of imprinted genes is controlled by posttranslational modifications of histones [5], non-coding (nc) RNAs [13,14,15] and chromatin conformation [16]. The integration of these different epigenetic marks with standard mechanisms of transcription and gene regulation (such as the use of alternative promoters) results in a fine-tuned control of imprinted gene expression in time and space, as exemplified for *Grb10*. In non-neural tissues, *Grb10* is maternally expressed as it is transcribed from its major promoter that is active only on the maternally inherited chromosome. By contrast, in neural tissues, *Grb10* is transcribed from at least three alternative promoters that are only active on the paternal chromosome [17,18,19,20]. These alternative promoters are situated in the *Grb10* ICR. On the maternal chromosome, the ICR is methylated and enriched for repressive histone marks [19,21]. By contrast, on the paternal chromosome, *Grb10* ICR is unmethylated and contains bivalent histone marks that are made of both H3K4me2 (permissive) and H3K27me3 (repressive) marks [21]. The paternal allele-specific bivalent mark on *Grb10* is maintained in tissues where there is no paternal expression while in the neural lineage the paternal repressive histone mark is resolved and paternal expression of *Grb10* increases [21]. Chromatin folding plays also a major role in the regulation of imprinted gene expression as exemplified at the *Igf2/H19* locus where chromatin conformation is intimately linked to access to shared enhancers [16,22,23,24,25]. ncRNAs are also central in the control of imprinted gene expression in time and space [13,26]. For example, *Ube3A*, which encodes an ubiquitin ligase involved in synaptic physiology [27,28,29] and whose function is lost in Angelman syndrome [30], is maternally expressed in the hippocampus while it is biallelic in the liver [29,31]. This is due to the presence in the hippocampus while absence in the liver of an antisense RNA transcript, *Ube3a-ATS* that cis-represses *Ube3A* paternal allele [32,33]. Finally, microRNAs may also regulate imprinted expression [8]. For instance, the paternally expressed *Rtl1* mRNA is a predicted target of several micro-RNAs processed from the *anti-Rtl1* RNA transcribed from the maternal allele [34].

## 2. The Brain Imprintome: How Many Imprinted Genes in the Brain? Transcriptome-Wide Identification of Imprinted Genes with Bulk RNA-seq

Imprinted genes play important roles in brain development and function [35,36]. Accordingly, genetic or epigenetic alterations that lead to altered imprinted gene expression cause several human brain disorders [6,35,37,38]. These imprinting brain diseases often have multifaceted clinical signs. For example, patients with Angelman syndrome suffer from seizures, cognitive impairment, hyperactivity, sleep disturbance and limited speech. This suggests that *Ube3a*, the gene affected in Angelman disease [39], ensures different functions in different brain regions or cell types. This was tested by experiments where *Ube3a* was deleted in specific groups of neural cells using tissue-specific knock-out mice. Loss of *Ube3a* in GABAergic neurons causes electroencephalogram abnormalities and increases susceptibility to seizures [40,41] and *Ube3a* loss in tyrosine hydroxylase-expressing neurons modifies reward-seeking behavior [42]. By contrast, *Ube3a* loss in glutamatergic neurons has no obvious effect [41]. The latter study was performed with *Ube3a**^m−/p+^*** mice (matKO mice), considering that only the maternal allele is expressed in the WT situation. However, *Ube3a* can be expressed at various degrees of parent-of-origin-dependent expression, ranging from a strict monoallelic maternal expression to biallelic expression depending on tissues [29,31]. It is, therefore, possible that some paternal expression compensated for the absence of the maternal allele in glutamatergic neurons. In addition, cell-type specific imprinting (also known as non-canonical imprinting [36]) was previously reported for other imprinted genes, including for *Dlk1* that is paternal in most cell types while it is biallelic (relaxed) in neural stem cells [43]. *DLK1* is an imprinted gene that is overexpressed in Kagami-Ogata syndrome and whose expression is lost in Temple syndrome, two imprinting disorders that affect the brain [44].

**Figure 1:**
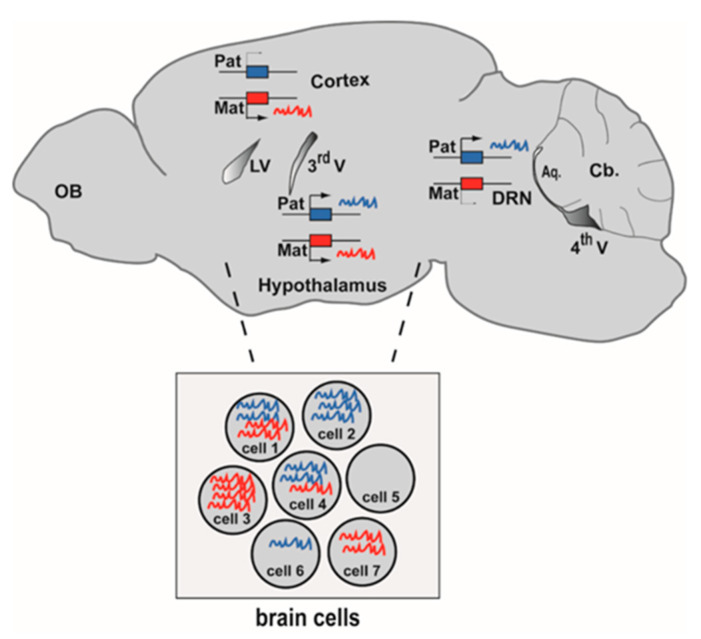
Genomic imprinting at tissue and cell resolution in the brain. Imprinted genes are differentially expressed from the maternal allele (red) and the paternal allele (blue). In this example of tissue-specific imprinting, an imprinted gene is maternally expressed in the cerebral cortex, paternally expressed in the dorsal raphe nucleus (DRN) while it is biallelic in the hypothalamus (sagittal view of a mouse brain at P56). The inset illustrates cell-specific imprinting: seven individual brain cells express different levels of RNAs of maternal and paternal origins. Aq: Cerebral aqueduct. Cb: Cerebellum. LV: lateral ventricle. OB: Olfactory bulb. 3rd V: Third ventricle. 4th V: Fourth ventricle.

*Cdkn1c* is an imprinted gene whose function is lost in Beckwith-Wiedemann patients [45,46], who frequently show brain abnormalities [47]. *Cdkn1c* was long considered to be exclusively expressed from the maternal allele in the brain. Recently, *Cdkn1c* was shown to be also expressed from the paternal allele in the developing cortex [48,49,50]. *Cdkn1c* paternal expression is much weaker than maternal expression but is above technical noise. Importantly, the development of the cerebral cortex is affected in *Cdkn1c^m+/p-^* (patKO) [49,50], demonstrating that the paternal allele plays a physiological role. Collectively, these examples illustrate that we need to define the parent-of-origin-dependent expression of imprinted genes in the normal and in the pathological brain at the levels of tissue, cell type and cell (Figure 1).

Tissue-specific and cell-type specific imprinting have been long recognized [51,52], as illustrated for *Gnas* [53], *Kcnq1* [54] and *Ube3a* [55]. One important question at that time was: How many imprinted genes show tissue-specific imprinting?

In 2008, Babak and co-workers [56], shortly followed by Wang and co-workers [57], paved the way by performing RNA-seq experiments on F1 hybrid mice. Taking advantage of parental strain-specific SNPs (Single Nucleotide Polymorphisms), they infer the parental origin of expression at the genome and organism levels. Hence, these authors identified the set of transcripts displaying parent-of-origin-dependent expression, what is sometimes called the ‘imprintome’. Since these landmark studies, many laboratories applied the same experimental strategy to search for genes with parent-of-origin-dependent expression, including in non–mammalian species. This notably confirmed previous estimates that there is no gene with parent-of-origin-dependent expression in the non-mammalian vertebrate *Gallus gallus domesticus* [58] while some in the angiosperm *Arabidopsis thaliana* [59].

Detailed maps of tissue-specific allelic expression were recently obtained by performing RNA-seq on more than 30 tissues of F1 hybrid mice [60,61] and in human [61]. The highest numbers of genes with parent-of-origin-dependent expression were detected in the tongue, the placenta, and the brain [60,61].

Bulk RNA-seq was also largely applied to specific brain regions, mostly isolated from the normal mouse brain (see Table 1). By contrast, data on the pathological brain are parsimonious. A recent report suggests that there is no parental bias of expression in schizophrenia patients [62]. The current consensus is that there are ~50–100 genes with parent-of-origin-dependent expression in the mammalian brain depending on regions, developmental stages, and methods, including statistical methods [60,61,63,64]. This number is rather close to earlier estimates made by Morison and co-workers who listed 83 imprinted genes in 2005 [10]. Not surprisingly, most of the novel imprinted genes identified using RNA-seq screens in human and mouse tissues or cultured cells are found at the border of previously identified imprinted loci and their parental bias is incomplete [60,61,65,66,67,68]. It is important to note that there is no gold standard statistical method to state whether a gene displays or does not display a parental bias of expression using RNA-seq on F1 hybrids. From a survey of current literature, we propose a list of six requirements. Table 1 shows how these requirements are met by 10 RNA-seq experiments performed on the brain of F1 hybrid mice.

**Figure 2:**
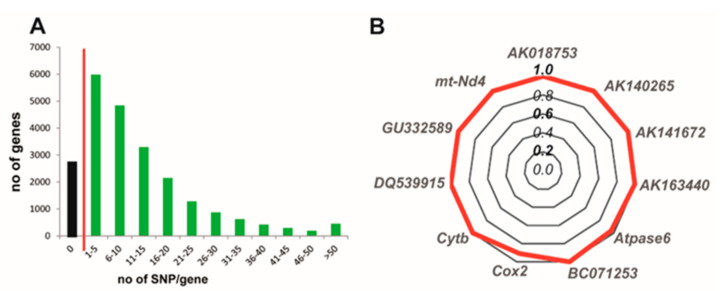
The number of SNPs in F1 hybrid mice and the confirmation of the maternal origin of mitochondrial transcripts are important steps when using RNA-seq to determine the imprintome. (**A**) Number of searchable genes in F1 hybrids generated by crossing C57BL/6 with JF1 mice. 20,426 genes out of 23,237 annotated genes have at least one exonic SNP. The red bar separates genes that contain or do not contain SNP. (**B**) Mitochondria reads are useful internal controls to check for cross direction in hybrids. RNA-seq was performed on cerebral cortex at P0 from reciprocal C57BL/6 × JF1 crosses. The radar chart shows the ratio of maternal/total number of reads for 11 mitochondrial genes. Since mitochondrial DNA is maternally inherited, reads should be maternal and ratio number of maternal reads/total number of reads should be 1. This is the case here with the exception of Cox2 whose ratio is 0.9. Please note that all of these 11 mitochondrial genes had more than 5 C57BL/6-JF1 SNPs. Number of mitochondrial reads were retrieved from samples published in [48].

The six requirements to perform RNA-seq on F1 hybrid mice are as follow:

**1. Reciprocal crosses to discriminate parent-of-origin-dependent expression from strain bias** [73]. The first matter when designing RNA-seq experiments on hybrid mice is certainly the choice of the two parental strains. These two strains must be sufficiently divergent so that the genome of the F1 hybrid progeny contains enough SNPs that will be informative for the parental origin of transcripts. Not surprisingly the number of SNPs is variable when comparing F1 hybrids generated by crossing 18 mouse common strains [74]. For example, there are 12,508,968 genomic SNPs between C57BL/6J and of *M. m. molossinus* Japanese Fancy (JF1)/Ms, which are two divergent mouse strains. In the (C57BL/6J × JF1) F1 hybrid, 20,426 genes out of 23,237 annotated genes (88%) have at least one SNP and 72% have more than 5 SNPs in their exons (Figure 2A). To interrogate the genes with no SNP, a third mouse strains can be introduced in the mating scheme.

The choice of the parental strains also has a major impact on subsequent steps, including reads alignment. RNA-seq reads need to be aligned to a hybrid reference genome so that there are no alignment penalties for the non–reference allele [75]. Indeed, if C57BL/6 genome is used as reference, reads originating from the C57BL/6 parent are favored over reads from the other parental strain. To limit this bias, reads are usually aligned to an artificial genome where SNP positions are replaced by N (i.e., all four possibilities in the IUPAC code), or better, by the IUPAC letter corresponding to the two possibilities associated with the SNP (see [48] for an example). In addition, indels and duplications, are known to influence read alignment but they are rarely taken into account.

Another important parameter is the choice of the RNA-sequencing pipeline. Guidelines can be found at the ENCODE project website (https://www.encodeproject.org/).

Most of the time, RNA-seq is performed on total RNAs or Poly A+ samples, which prevents from determining the parental origin of small RNAs, including microRNAs that represent a major class of imprinted RNAs [8].

Once reads are aligned and assigned to one or the other parental strains, an internal control that can be used to check for the direction of parental bias is mitochondrial (mt) transcripts. Indeed, since mtDNA is exclusively (or predominantly [76]) transmitted by the mother, mitochondrial reads should be quantified as maternally expressed, as shown in Figure 2B. Additional useful controls are X-linked genes that should be quantified as biallelic due to random X-inactivation in females [1].

In humans, discriminating parental from strain bias through the use of reciprocal crosses are obviously not feasible. To eliminate allele-specific expression that is due to cis-regulation of genetic variants, Babak and co-workers reasoned that biased expression caused by genetic variants would lead to a consistent expression bias from the same allele in heterozygous individuals, whereas for imprinted genes no particular allele should be favored [61]. Applying this strategy to 1687 RNA-seq samples from 178 individuals, they confirmed 63 imprinted genes and identified 17 novel candidates. Interestingly, 17 imprinted genes were confirmed using lymphocytes from a family with three generations, so that the direction of allele-specific expression can be followed over generations [61]. Similarly, Santoni and co-workers excluded cis-expression quantitative trait loci with recurrent strong expression from their analysis of human RNA-seq data [77]. To detect parent-of-origin biases in gene expression, Jadhav and co-workers combined allelic analysis of RNA-seq data with phased genotypes in family trios [65]. A detailed map of human imprinting in the Icelandic population was obtained by combining RNA-seq, whole genome bisulfite sequencing and parent-of-origin phased haplotypes [78].

**2. Statistical method**. The experimental design should include an appropriate number of replicates from the start of the pipeline so that statistics are powerful enough for the analysis. The importance of the choice of the statistical framework is illustrated by the controversy that arose when an unexpectedly high number of imprinted loci was found in 2010 [70], which was much higher than the number reported two years before (albeit on different samples) [56]. This number was later found to be exaggeratedly high mostly because of incorrect statistical analysis [63,64]. A comprehensive comparison of most statistical methods employed so far to analyze RNA-seq data obtained on hybrid mice can be found in [74].

**3. Concordance between individual SNPs**. Logically, SNPs from the same transcript should display the same parental bias. Statistical tests must be used to define the threshold number of SNPs that must be concordant. To our knowledge, most RNA-seq experiments performed so far have not taken SNP concordance into account (Table 1).

**4. Strand-specific-RNA-seq to distinguish between overlapping transcripts**. Some imprinted genes are transcribed from the two parental alleles on opposite directions. For example, at the *Dlk1-Dio3* locus on mouse chromosome 12, two maternally expressed microRNAs are transcribed antisense to the paternally expressed retrotransposon-like gene *Rtl1* gene [34]. Strand-specific-RNA-seq is therefore obligatory to allocate reads originating from two overlapping antisense transcripts. However, strand-specific-RNA-seq is not commonly employed (Table 1).

**5. Confirmation of RNA-seq data by other techniques interrogating parental origin**. It is necessary to confirm the allele-specific expression data obtained by RNA-seq by complementary methods that use SNPs to interrogate the parental origin of transcripts such as pyrosequencing, iPLEX Sequenom, Sanger sequencing of PCR products and Restriction Fragment Length Polymorphism (RFLP) (Table 1).

**6. Corroboration of RNA-seq data with additional epigenetic marks**. It is interesting to complement allele-specific expression data with epigenetic marks that are associated with genomic imprinting such as DNA methylation and histone modifications (Table 1). For example, we found a high concordance of parent-of-origin-dependent expression (using RNA-seq) and parent-of-origin DNA methylation at DMRs (using Reduced Representation Bisulphite Sequencing, RRBS) in the developing mouse cerebral cortex [48]. Allelic expression is associated with differential enrichment of H3K27ac [60], a histone mark known to be enriched at active enhancers [79]. The repressive H3K9me3 mark is enriched on the silenced allele of *Plagl1* and *Meg3* while there is no enrichment for the active H3K9ac mark on the expressed allele [69].

## 3. Monoallelic Expression of Imprinted Genes at Single-Cell Resolution: Insights from Molecular Probes, Genetic Reporters and scRNA-seq

Genes with an incomplete parental bias are frequently observed in bulk RNA-seq experiments, notably in brain tissues [48,60,61,73]. Two alternatives can explain this incomplete parental bias: these genes could be either equally biased in all the cells (so that the sum of parental bias in every cell reflects the average bias measured in the whole tissue), or these genes could be monoallelic in some cells and biallelic in other cells (Figure 1). An analysis at single-cell (sc) resolution is required to discriminate between these two possibilities.

### 3.1. Probes

SNP-FISH is a situ hybridization technique that uses fluorescent probes to detect RNA molecules in a SNP-specific manner [80]. SNP-FISH with two probes, one specific for the C57/BL6J sequence and another one specific for the *Mm castaneus* sequence, revealed that *H19* is maternal in every mouse embryonic fibroblasts and heart cells [81]. By contrast, in fibroblasts with a paternally inherited mutation at the insulator sequence, *H19* is biallelic in 77% of cells (while it remains monoallelic in 23% of cells). Because all mutant fibroblasts are supposed to have the same genotype, epigenetic difference is the likely cause of *H19* mosaicism. In addition, *Igf2* is only expressed in cells where *H19* is predominantly maternal, supporting the established model of an insulator that prevents enhancer activity at this locus [82].

RNAscope on nascent RNAs is another microscopy technique that was used to study imprinted gene expression at the cell level [69]. With RNAscope, monoallelic and biallelic expression are visualized as one or two dots, respectively [69]. *Ago2*, a gene detected with an incomplete parental bias in RNA-seq experiments, is monoallelic in some cells while it is biallelic in other cells [69], suggesting that *Ago2* displays cell-specific imprinting in brain cells [69]. However, positive controls, including *Maoa*, a X-linked gene expected to be monoallelic due to X-inactivation and *Syn2*, a gene expected to be biallelic, were not quantified as strictly monoallelic and biallelic [69]. This variability might be due to technical reasons, including the difficulty to quantify dots in brain slices, to mRNAs expressed at low level and to transcriptional bursting, a known source of allelic imbalance [83,84].

### 3.2. Reporters

Reporters are a complementary approach to visualize cell-type specific imprinting. Venus and Tomato reporter genes were inserted into *Dlk1*, resulting in color-coded parental expression. In *Dlk1^MatTom/PatVen^* mice, maternal expression is red, paternal expression is green and biallelic expression is yellow [85]. To our knowledge, only cell sorting experiments were performed with this tool and it will be interesting to obtain fluorescent images at cell resolution in brain slices. Bonthuis and co-workers [86] have developed transgenic mice that allow following the parental expression of *Ddc*, a gene that encodes for Dopa decarboxylase, an enzyme that is required for the synthesis of dopamine and that was previously found with a parental bias in many organs, with a maximal bias in the hypothalamus [69]. Using hypothalamic slices from *Ddc ^eGFP/V5^* (and reciprocal *Ddc ^v5/eGFP^* mice), *Ddc* was found biallelic in most cells and preferentially maternal in some cells [86].

### 3.3. Single Cell RNA-seq

**Figure 3:**
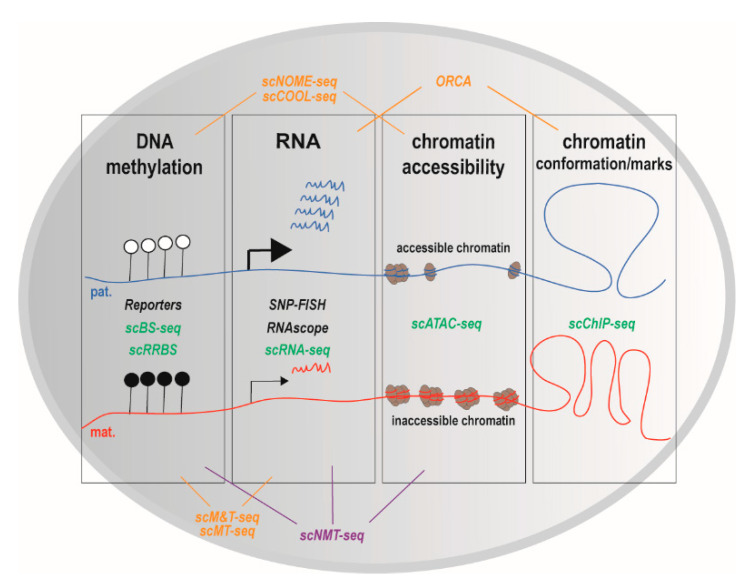
Methods that interrogate genomic imprinting at cell resolution. Several features of genomic imprinting, including DNA methylation, expression (RNA), chromatin accessibility, chromatin conformation and histone marks, can be interrogated in a parent-of-origin-dependent manner using single-cell methods. Locus and gene-specific methods are written in *black*. Genome-wide and transcriptome-wide methods are colored as follows: omic methods that interrogate a single feature are in *green*, omic methods that interrogate two features are in *orange*. scNMT-seq *(purple*) simultaneously interrogates three features (DNA methylation; transcription and chromatin accessibility (by labeling accessible chromatin that is nucleosome-depleted). In the pictured imprinted locus, DNA is methylated on the maternal chromosome only and RNAs are principally produced from the paternal allele (as observed for imprinted loci with maternal DMRs), and the chromatin accessibility and conformation differ between the two parental alleles. ORCA: Optical Reconstruction of Chromatin Architecture; sc: single cell; seq: sequencing; scATAC-seq: sc Assay for Transposase-Accessible Chromatin-seq; scBS-seq: sc Bisulfite-seq; scChIP-seq: sc Chromatin Immunoprecipitation-seq; scCOOL-seq: sc Chromatin Overall Omic-scale Landscape-seq; scMT and scM&T: sc-Methylation & Transcription-seq; scNMT-seq: sc Nucleosome, Methylation and Transcription-seq; scNOME-seq: sc Nucleosome Occupancy and Methylome-seq; scRRBS: sc Reduced Representation Bisulfite-seq; SNP-FISH: Single Nucleotide Polymorphism-Fluorescent In Situ Hybridization.

In addition to imaging techniques that investigate a limited number of genes, single-cell RNA-sequencing (scRNA-seq) (Figure 3) is emerging as a powerful technique to get insights into cell-specific imprinting transcriptome-wide. The first scRNA-seq study on hybrid mice (CAST/EiJ × C57BL/6J) revealed that random monoallelic gene expression is widespread in individual embryonic cells [87]. scRNA-seq on hybrid mice was also used to get insights into the expression of olfactory receptors in individual mouse olfactory neurons [88]. To our knowledge, the first scRNA-seq study that yielded insight into cell-specific imprinting was performed in 2017 by Santoni and co-workers [77]. In 1084 primary human fibroblasts, they detected 50 known imprinted genes, from which 45 had a parental bias and five were biallelic [77]. Their screen also identified nine imprinted candidates. Interestingly, these nine candidates were undetectable in bulk RNA-seq. Finally, they also found 33 additional genes with parental imbalance but authors estimate that more family trios are needed to confirm that these genes are truly imbalanced [77]. Recently, Bonthuis and co-workers [86] investigated the repertoire of imprinted genes by reanalyzing scRNA-seq data of hypothalamus from F1 C57B6 × DBA2 hybrid mice—single cross direction—that were produced by others [89]. This set of data comprises 45 clusters, i.e., 45 groups of cells with a close transcriptional signature—a proxy to cell identity—here 11 non-neural and 34 neural hypothalamic subtypes. Bonthuis and co-workers found that imprinted genes are differentially expressed (enriched) in these clusters [86]. However, there was no information on genomic imprinting at cell resolution.

Laukoter and co-workers performed allelic scRNA-seq on mouse cortical cells, providing important insights on cell-specific imprinting [90]. On 223 B6xCAST and 181 CASTxB6 cortical cells that passed their quality filters, the parental bias was found to be uniform for 20/25 imprinted genes [90]. As an example, *Plagl1* was 100% paternal in all of the cells from the five cell types resulting from their clustering (nascent and mature projection neurons, astrocyte progenitors, mature astrocytes and oligodendrocytes) [90]. By contrast, for 5/25 imprinted genes, there was cellular heterogeneity in the parental bias (i.e., cell-specific imprinting). For instance, *Grb10* was 100% maternal in astrocyte progenitors while it was 100% paternal in mature astrocytes [90], suggesting that there is a switch in *Grb10* promoter usage during astrocyte differentiation, as previously described with bulk samples [17,18,19,20]. However, for many imprinted genes the number of informative cells is low or insufficient [90]. Notably, *Dlk1*, which was previously described to be either monoallelic or biallelic in different brain cell types [43] could not be analyzed. Technically, Laukoter and co-workers have used Smart-seq2, which generates full length cDNAs and permits to obtain information on genes using alternative transcription start sites [91] (such as *Grb10*). On the other hand, Smart-seq2 uses oligo(dT) primers, which prevents from obtaining information on a subset of imprinted gene products such as miRs and non-polyadenylated lncRNAs [91]. In addition, Laukoter and co-workers observed that the two biallelic control genes (*Ncam1* and *Fgfr2*) were monoallelic in some cells [90], which presumably reflects transcriptional bursting [84].

This example illustrates that it might be difficult to distinguish between the stable monoallelic expression of imprinted genes and the transient monoallelic expression due to transcriptional bursting. In addition, one important remaining question is how to differentiate at the cell level between the deterministic monoallelic expression that characterizes imprinted genes and the stochastic monoallelic expression of genes with RMAE. The well-known imprinted MAE depends on DNA methylation marks that are differentially deposited in the maternal and paternal germlines at ICRs [4,6,51]. By contrast, for RMAE the stochastic choice of the expressed allele is not well explained. RMAE was studied in clonal cell populations and different genes with RMAE are observed in different tissues [2,3,87,92,93]. RMAE affects ~2% of autosomal genes with various functions; many of them encode cell-surface proteins. The fact that these genes can be expressed either biallelically or monoallelically from the maternal or the paternal allele is thought to increase diversity among a cell population [2,3]. In addition, mitotically stable RMAE has to be distinguished from transient RMAE due to transcriptional bursting and from biased allelic expression due to DNA sequence polymorphism. Limiting concentrations of transcription factors have been proposed as one of the mechanisms involved in RMAE [2,3]. Epigenetic marks such as histone methylation were observed at promoters of RMAE genes (H3K4me2/3 on the active allele and H3K9me3 on the inactive allele), but RMAE does not seem dependent on DNA methylation in contrast to imprinted MAE [2,3].

As brain cells work together, it is important to preserve information about their spatial organization into specialized neural networks. In this context, the identity and position of cell types were mapped in sections of preoptic hypothalamus of mice by combining scRNA-seq with MERFISH (multiplexed error-robust fluorescence in situ hybridization) [94]. In addition, by identifying RNAs that were co-expressed with the marker of neuronal activity c-Fos, authors retrospectively mapped the cells that compose neural networks activated during specific tasks [94]. In the context of genomic imprinting, scRNA-seq combined with a modified MERFISH that uses SNP-FISH probes could be used to map the imprintome in individual brain cells while preserving spatial information about their location. In addition, we could imagine identifying the parental expression of imprinted genes in the neural networks activated during tasks that are associated with genomic imprinting functions such as lactation and parental care. This might be a first step toward determining whether or not cell-specific imprinting has a physiological impact on brain function [36].

## 4. Single-Cell Epigenomics and Single-Cell Multi-Omics: Promising Approaches to Comprehend Genomic Imprinting at Cell Resolution

In addition to single-cell techniques that interrogate parent-of-origin-dependent expression, single-cell technologies also permit to quantify additional features of genomic imprinting, including DNA methylation, histone modifications and chromatin conformation [95] (Figure 3).

### 4.1. DNA Methylation

Several methods were developed to measure DNA methylation in single cells. Some methods rely on genetic constructs that report on the methylation status of endogenous imprinted promoters [96,97]. Other methods such as scNOME-seq [98] (nucleosome occupancy and methylome sequencing [99]), sc bisulfite sequencing (scBS-seq) [100] and scRRBS [101] take advantage of next generation sequencing to interrogate DNA methylation genome-wide (Figure 3). scRRBS provides information on the methylation status of up to 1.5 million individual CpGs in single mESCs (mouse Embryonic Stem Cells), compared to 2.5 million informative CpG for bulk samples [101]. As expected, compared to scRRBS, scBS-seq better covers the methylome (up to 48.3% of genomic CpGs) [100]. The snapshot resulting from the merge of individual methylation profiles resemble the methylation profile obtained with bulk samples [100,101]. Of interest in the context of genomic imprinting, the imprinted *Plagl1* locus was methylated in each of 12 oocytes, as expected for a maternal DMR [100]. Both scRRBS and scBS-seq revealed inter-individual variation in DNA methylation of mESC [100,101], which presumably reflects variations in their pluripotency. To our knowledge, these methods have not been applied to genomic imprinting in brain cells.

### 4.2. Chromatin Modifications and Conformation

Several methods were also developed to quantify chromatin modifications and chromatin conformation in single cells (reviewed in [95,102], and Figure 3). This includes scChIP-seq that quantifies histone modifications [103], ORCA (Optical Reconstruction of Chromatin Architecture) that visualizes DNA folding [104] and scATAC-seq (Assay for Transposase-Accessible Chromatin)-seq that interrogates chromatin accessibility, a proxy to identify regulatory regions that are potentially exploited in a cell. scATAC-seq was performed on hybrid ESC and neural progenitors [92], providing insights into the landscape of monoallelic DNA accessibility. Unfortunately, imprinted genes were filtered out [92]. Using NOME-seq, which interrogates nucleosome occupancy (in addition to DNA methylation), Kelly and co-workers found that the promoters of imprinted genes are enriched in divergent chromatin alleles, meaning that the two alleles exist in two different chromatin states [99], as expected for imprinted genes [25]. Data mining of scNOME-seq data [98] should inform whether this also applies to individual cells.

### 4.3. Multi-Omics

Currently, one major challenge is to simultaneously measure several features of genomic imprinting in the same cell. Several single cell multi-omics approaches that interrogate up to three features (transcriptome, DNA methylation, and chromatin organization) have been developed (reviewed in [95,102], and Figure 3). sc Methylation & Transcription-seq (scM&T-seq) simultaneous quantifies transcriptome (using RNA-seq) and methylome (using BS-seq) in single cells (isolated by flow cytometry) [105]. Importantly, scM&T-seq gives results in agreement with data obtained with scBS-seq [105]. scM&T-seq experiments have revealed that the correlation between methylome and transcriptome varies between individual mESCs [105]. As previously observed with bulk samples, there is an inverse correlation between the levels of expression and of DNA methylation for a series of pluripotency factors [105]. scMT-seq [106] is a quite similar approach to scM&T-seq (scMT-seq is based on scRRBS while scM&T-seq is based on scBS-seq). scMT-seq revealed a positive correlation of gene body methylation with gene expression only for genes that contain a CpG island in the promoter in individual dorsal root ganglion neurons [106]. Guo and co-workers have developed scCOOL-seq (Chromatin Overall Omic-scale Landscape Sequencing) [107], which interrogates both DNA methylation and chromatin accessibility (Figure 3). Interestingly, scCOOL-seq on hybrid cells revealed that maternal and paternal chromosome have different dynamic of DNA methylation and chromatin remodeling in individual cells during early developmental stages (from zygote to blastocyst stages) [107]. In addition, as expected, Guo and co-workers have observed that *Impact* ICR was only methylated on the maternal chromosome [107].

scM&T-seq was recently upgraded to additionally quantify chromatin accessibility by labeling accessible chromatin that is nucleosome-depleted (Figure 3). This upgraded version of scM&T, called scNMT-seq (for sc nucleosome, methylation, and transcription) takes advantage of a GpC methyl transferase that labels accessible chromatin, followed by bisulfite treatment and DNA-sequencing (as NOME-seq does [99]), and RNA-sequencing [108]. Argelaguet and co-workers have applied scNMT-seq to individual mouse embryonic cells at the onset of gastrulation (from E4.5 to E7.5), when cells exit pluripotency to form the three primary germ layers [109]. Interestingly, they found that the epigenetic landscape of ectodermal cells is already set in the early epiblast whereas for mesoderm and endoderm lineages, there is a vast epigenetic rearrangement at enhancers (with a key role for TET-driven demethylation), concomitant with an increase in chromatin accessibility [109]. Please note that one drawback of NOME-seq derived methods such as scCOOL-seq and scNMT-seq is that some CpG sites cannot be interrogated.

To conclude, sc-omics and sc-multi-omics techniques provided important insights on how the different epigenetic layers and transcriptome interact during cell fate decision, a fundamental question in developmental biology [110]. However, sc-omics experiments brought limited information on genomic imprinting at cell resolution, with the exception of two recent studies [77,90]. It will be worth data mining previously performed sc-omics experiments and to perform sc-omics experiments on a hybrid background. sc-omics approaches that interrogate the phenotype of individual cells, such as Perturb-seq [111], should also bring important insights into imprinted genes function. Finally, sc-omics approaches are limited by important cost per-cell, low signal to noise ratio and partial coverage of transcriptome, methylome and/or genome [102]. A practical guide to most of the single-cell methods that we have mentioned here, which includes detailed protocols ranging from tissue preparation to bioinformatics analysis of sc-data, has been recently compiled in a comprehensive book [112].

## 5. Concluding Remarks

Genomic imprinting is central to brain homeostasis, as exemplified by genomic imprinting diseases that affect brain development and that have long term repercussions on brain function. A better understanding of the roles of genomic imprinting in the brain calls for the identification of imprinted genes in brain tissues, cell types and individual cells. Bulk RNA-seq on F1 hybrid mice associated with techniques investigating other features of genomic imprinting, including parent-specific DNA methylation and histone modifications, provided valuable insights into tissue-specific genomic imprinting. However, our knowledge is still fragmentary for pathological situations, especially those affecting development of the human brain. Single-cell technologies, including scRNA-seq and imaging experiments recently revealed the existence of cell-type specific imprinting, whose functional impact is unknown. Finally, sc-omics techniques are expected to yield important information on genomic imprinting signature and function in individual brain cells.

## Figures and Tables

**Table 1:** Features of imprintome studies performed on mouse brain samples.

Study: First Author Name, Reference	Brain Region	Mouse Strains	Reciprocal Crosses	Replicates	Concordance of SNPs	Strand Specific RNA-seq	Validation by Other Methods	Additional Epigenetic Marks
Andergassen, [60]	whole brain	FVB × Cast	Yes	Yes	Yes	Yes	No	allele-specific ChIP (H3K27ac)
Babak, [61]	13 brain parts	C57BL/6 × Cast	Yes	Yes	Yes	Yes	Pyrosequencing	DNA Methylation
Bonthuis, [69]	arcuate and dorsal raphe	C57BL/6 × Cast	Yes	Yes	Yes	No	Pyrosequencing, RNAscope	allele-specific ChIP (H3K9ac and H3K9me3)
Bouschet, [48]	cerebral cortex	C57BL/6 × JF1	Yes	Yes	No	Yes	Sanger/RFLP	DNA Methylation
DeVeale, [63]	whole brain	C57BL/6 × Cast	Yes	Yes	Yes	Yes	Pyrosequencing	No
Gregg, [70]	Cortex and hypothalamus	C57BL/6 × Cast	Yes	Yes	No	No	iPLEX Sequenom	No
Lin, [71]	3 cell types of visual cortex	C57BL/6 × Cast	Yes	No	No	No	Sanger	No
Lorenc, [72]	hypothalamus	WSB × PWD	Yes	Yes	Yes	No	Pyrosequencing	No
Perez, [66]	cortex, hypothalamuscerebellum	C57BL/6 × Cast	Yes	Yes	No	No	Pyrosequencing	No
Wang, [57]	Whole brain	PWD × AKR	Yes	Yes	No	No	Sanger Pyrosequencing	No
